# Designer antiandrogens join the race against drug resistance

**DOI:** 10.7554/eLife.00692

**Published:** 2013-04-09

**Authors:** Jatinder S Josan, John A Katzenellenbogen

**Affiliations:** 1 **Jatinder S Josan** is at the Department of Chemistry, University of Illinois at Urbana-Champaign, Urbana, United Statesjsjosan@illinois.edu; 2 **John A Katzenellenbogen** is at the Department of Chemistry, University of Illinois at Urbana-Champaign, Urbana, United Statesjkatzene@illinois.edu

**Keywords:** prostate cancer, androgen receptor, drug resistance, Human, Mouse

## Abstract

By using in silico models of the complexes formed by analogues of a cancer drug and its receptor, it may be possible to strategically redesign existing drugs and win the race against mutations that lead to drug resistance in prostate cancer.

**Related research article** Balbas MD, Evans MJ, Hosfield DJ, Wongvipat J, Arora VK, Watson PA, Chen Y, Greene GL, Shen Y, Sawyers CL. 2013. Overcoming mutation-based resistance to antiandrogens with rational drug design. *eLife* **2**:e00499. doi: 10.7554/eLife.00499

**Image** A model of the interaction between the prostate cancer drug enzalutamide and the androgen receptor

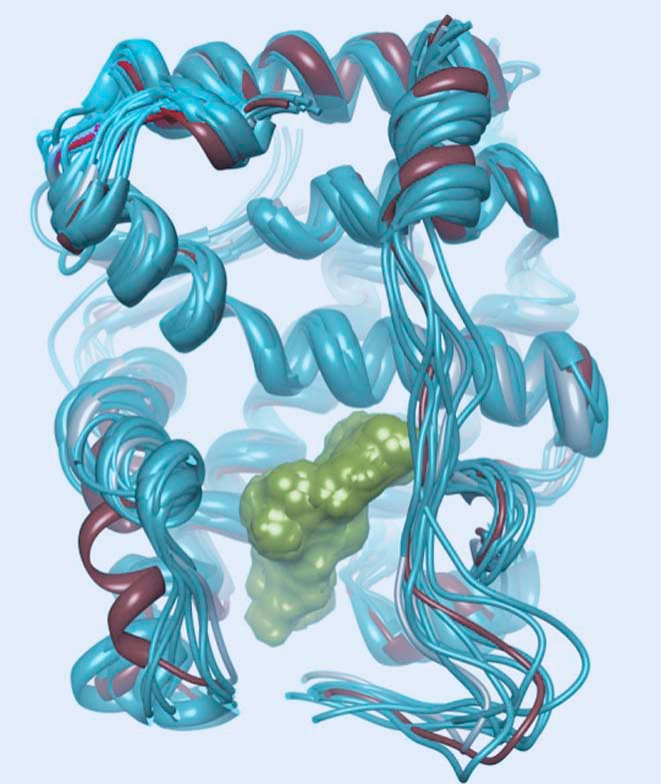

In Lewis Carroll's *Through the Looking-Glass*, the Red Queen reveals to Alice that in her world ‘it takes all the running you can do, to keep in the same place. If you want to get somewhere else, you must run at least twice as fast as that!’. The predicament in prostate cancer is much the same: researchers and clinicians alike are forced to keep running merely to stay still, because the receptors targeted by prostate cancer drugs are continually undergoing mutations that prevent the drugs from working. Now, in *eLife*, Minna Balbas, Charles Sawyers and their colleagues at the Memorial Sloan-Kettering Cancer Center and the University of Chicago may have found a way to run twice as fast, by using a novel approach to redesign drugs to restore their clinical efficacy (Balbas et al., 2013).

Prostate cancer is driven largely by male hormones, otherwise known as androgens, acting through the androgen receptor (Figure 1A). Consequently, the majority of cases can be treated by depriving the tumors of androgens: this is achieved by suppressing the production of androgens and by administering antiandrogen drugs, such as flutamide and bicalutamide (Figure 1B). By binding to the same site on the receptor as endogenous androgens, these drugs—which are known as receptor antagonists—prevent the hormones from activating the receptor. With time, however—sometimes only a matter of months—the cancer returns in a form resistant to these therapies, termed ‘castration-resistant prostate cancer’. Resistance can arise through various means (Knudsen and Scher, 2009), but many cases result from mutation of the androgen receptor (e.g., Veldscholte et al., 1990; Hara et al., 2003; Figure 1C). This is because the gene encoding the androgen receptor is on the X chromosome, which means that men have only a single copy and therefore any change in the gene must be expressed in the protein.

**Figure 1.**
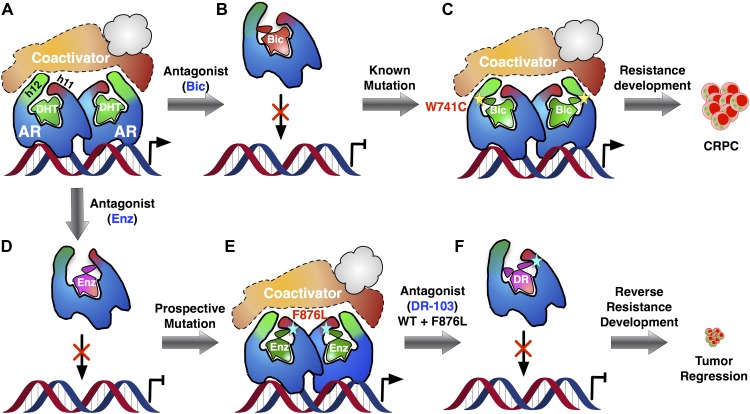
Addressing the development of resistance to antiandrogen drugs is an important challenge in prostate cancer research. (**A**) Prostate cancer is driven by male hormones (androgens) such as dihydrotestosterone (DHT) binding to the androgen receptor (AR). This enables proteins called coactivators to bind to the receptor, which can then act as a nuclear transcription factor, leading to growth of the tumor. (**B**) Conventional prostate cancer treatments, such as bicalutamide (Bic), are androgen receptor antagonists. By binding to the same site on the receptor as endogenous androgens, they prevent activation of the receptor. (**C**) Antiandrogen drugs fail when mutations in the androgen receptor change its structure in a way that allows the receptor to be activated, even when the drug is bound to it: this leads to the emergence of drug-resistant forms of prostate cancer (CRPC). The yellow star denotes the W741C mutation that makes the receptor resistant to Bic. (**D**) Enzalutamide (Enz) is a potent drug for treating prostate cancer, but its efficacy is also threatened by the emergence of drug resistance. (**E**) To tackle this problem, Balbas et al. took a prospective approach: They identified a new mutation (cyan star, F876L) that completely removed the therapeutic efficacy of Enz. (**F**) Then, guided by a model of the structure of the drug–receptor complex, they developed an alternative form of Enz (DR) that prevents activation of both wild-type and mutant receptors. Treatment with DR led to a marked reduction in prostate cancer cell proliferation and tumor growth, even for the L876 mutant that was resistant to Enz. Ligands are highlighted in green when the androgen receptor complex is activated and in red-mauve when the complex is inactivated.

Over the past few years, Sawyers and collaborators have brought a number of more effective antiandrogens to the clinic, most notably enzalutamide (Enz) (Figure 1D). This drug binds to the androgen receptor with high affinity and effects a more complete blockade of receptor signaling than earlier drugs, providing significant survival benefit in advanced castration-resistant prostate cancer (Scher et al., 2012). Nevertheless, cases of resistance to Enz are being encountered in the clinic (Efstathiou et al., 2012; Richards et al., 2012).

Balbas et al. dived directly into the drug resistance conundrum by using structural modeling to guide the development of new Enz analogues that are able to inhibit the mutant androgen receptors. They developed a system to monitor androgen receptor activity using flow cytometry, and then used random mutagenesis to produce mutant forms of the receptor. Treating cells containing these receptors with Enz, and repeatedly selecting those that survived, led to the emergence of one particular mutation—known as F876L—in which Enz activated the receptor (that is, Enz acted as an agonist). Enz stimulated the growth of cells containing this receptor, both in vitro and in tumor tissue grafts introduced into mice (xenografts) (Figure 1E). Even without prior mutagenesis, this same Enz-resistant genotype (and a closely related mutation called F876I) emerged spontaneously both in cells and in xenografts grown in the presence of Enz. Mutant drug-resistant forms of therapeutic targets typically no longer bind the drug that should inhibit them—a loss of function; by contrast, the mutant androgen receptor binds Enz six times more effectively than the wild-type receptor does, and responds to it as an agonist—a double gain of function.

So, what can be done to restore ‘lack of function’—that is, antagonism—to drugs faced with the F876L mutant receptor? Here, the use of molecular modeling to simulate receptor–drug complexes can provide valuable guidance. Balbas, Sawyers and co-workers modeled androgen receptors complexed with bicalutamide and related analogues, and evaluated the orientation of the drug within the complex. The results were instructive, revealing that Enz binds in an orientation different from that of bicalutamide (Figure 1B vs Figure 1D). The alternative mode of receptor antagonism favored by Enz has been observed before in the estrogen receptor, where it was termed ‘indirect antagonism’ (Shiau et al., 2002; Zheng et al., 2012). When Enz binds to the wild-type androgen receptor, an interaction between the drug and the F876 residue in the receptor appears to angle Enz in such a way as to block coactivator binding. However, modeling suggests that when Enz binds to the L876 mutant receptor, this interaction no longer occurs, and the receptor instead adopts a conformation in which Enz functions as an agonist (Figure 1E).

So, might it be possible to alter drug interaction with the L876 mutant receptor to restore antagonism? The investigators used their model of Enz bound to the L876 androgen receptor to guide modifications of the Enz structure, enlarging it in a specific direction until they identified an Enz analogue that induced an antagonist conformation in the L876 mutant (Figure 1F). Gratifyingly, these larger analogues, notably one known as DR103, proved to be effective antagonists of mutant as well as wild-type androgen receptors in both cellular and xenograft prostate cancer models.

It would be reassuring to have these computational models verified by crystallographic studies, although obtaining androgen receptor structures with antagonists has thus far proven difficult. Moreover, some open questions remain: are the F876L/I androgen receptor mutations clinically relevant, appearing in patients when resistance to Enz develops? Can the success of this approach be further validated by applying the same principle to bicalutamide, and designing analogues that restore antagonist efficacy against bicalutamide-resistant mutants? Will L876 mutant tumors treated with DR103 develop further androgen receptor mutations that then defeat the antagonism of these new analogues, requiring another lap in the Red Queen's race?

Thus, it remains to be determined whether this preemptive design of antiandrogens active on wild-type and prospective androgen receptor mutants will improve long-term survival in advanced castration-resistant prostate cancer. Finally, although genetic analysis has revealed a strong association between point mutations and resistance to antiandrogens, androgen receptor mutation does not account for most cases of castration-resistant prostate cancer (Taplin et al., 2003; Taplin and Balk, 2004). Thus, the potential utility of this approach rests on the development of suitable biomarkers and the identification of patients who could benefit from therapy directed to their particular androgen receptor genotype.

Prostate cancer is poised to enter a new era of personalized health care. The next breakthroughs in therapeutics will occur with an in-depth mechanistic understanding of the events underlying disease progression and the development of resistance. The approach taken by Balbas et al. provides a compelling paradigm for improving the durability of therapies, by strategically redesigning drugs to compensate for resistance arising from mutations in their targets. The same approach could also used to extend the effectiveness of other targeted therapies before resistance arises.
